# Different maturation cocktails provide dendritic cells with different chemoattractive properties

**DOI:** 10.1186/s12967-015-0528-7

**Published:** 2015-06-03

**Authors:** Chiara Massa, Carolin Thomas, Ena Wang, Francesco Marincola, Barbara Seliger

**Affiliations:** Institute of Medical Immunology, Martin Luther University Halle-Wittenberg, Magdeburger str. 2, 06112 Halle (Saale), Germany; Department of Transfusion Medicine, National Institute of Health Clinical Center, Bethesda, USA; Sidra Medical and Research Center, Doha, Qatar

**Keywords:** DC, Vaccination, Chemokines, Human, Innate immunity, T cells

## Abstract

**Background:**

Dendritic cells (DC) are currently implemented as immunotherapeutic strategy for the treatment of tumor patients based on their central role in the immune system. Despite good results were obtained in vitro and in animal models, their clinical use has provided limited success suggesting the requirement to optimise the protocol for their production.

**Methods:**

A cDNA array was performed on FastDC obtained from the differentiation of human peripheral blood monocytes stimulated with the clinical gold standard or with two alternative maturation cocktails combining interferon (IFN)γ and ligands for different toll like receptors (TLR).

**Results:**

A stronger modulation of the DC transcriptome with respect to immature DC was found in alternatively stimulated DC when compared to DC stimulated with the clinical gold standard. A major class of molecules differentially expressed using distinct DC stimulation protocols were chemokines. Validation of their differential expression pattern at the mRNA and protein level confirmed the secretion of inflammatory chemokines by the alternative DC. Functional analyses of the chemotactic properties of DC “wash out” supernatants highlighted the ability of alternative, but not of gold standard DC to efficiently recruit immune cells with a prevalence of monocytes. Effector cells belonging to the innate as well as adaptive immunity were also attracted and the interaction with alternative DC resulted in enhanced secretion of IFNγ and induction of cytotoxic activity. Using leukocytes from cancer patients, it was demonstrated that the monocyte-attracting activity targeted cells with an inflammatory phenotype characterised by high levels of HLA-DR expression.

**Conclusions:**

Despite other classes of immune modulatory genes differently expressed in the alternative DC require to be investigated and characterised regarding their functional consequences, the reduced maturation state and chemoattractive properties of the gold standard versus alternative DC clearly promote the necessity to change the clinically used maturation cocktail of DC in order to improve the outcome of patients treated with DC-based vaccines.

## Background

During the last two decades strategies have been developed to implement dendritic cell (DC)-based therapies for the treatment of cancer. Despite their successful usage in murine models, this approach demonstrated only a limited efficacy in the human setting, although the approval of sipuleucel-T (also called APC8015 or Provenge®) [1] from the Food and Drug Administration for the therapy of prostate cancer patients has provided new strength to further pursuing this path.

In order to improve the functionality of vaccine DC the established protocols have been modified by changing (1) the source of cells [2, 3], (2) the differentiation protocol when starting from monocytes or precursor cells [4–6] and (3) the composition of the maturation cocktails in order to provide all necessary functions to the DC [7–9].

In addition to providing all the required signals for a proper activation of effector cells, an important requirement of vaccine DC is their ability to “reach” the effector cells, mediated either by their own migration or by the recruitment of effector cells. Chemokines and their receptors exploit a central role in the correct homing of immune cells under homeostatic and inflammatory conditions [10]. Whereas the ability of vaccine DC to migrate toward secondary lymphoid organs has been deeply investigated, less attention has been paid to their chemoattracting properties.

Recently, we demonstrated that monocytes differentiated with the shortened 48 h-long Fast protocol [5] and stimulated with alternative (alt-) cocktails containing toll like receptor (TLR) ligands and interferon (IFN)γ (for full description see Table 1) displayed an enhanced ability to interact with innate and adaptive effector cells when compared to the gold standard cytokine cocktail-matured DC due to an enhanced expression of costimulatory molecules and secretion of interleukin (IL-)12p70 (see Table 2; Ref. [11]). In order to further dissect the differences of DC matured with the gold standard or the alternative cocktails, cDNA microarrays were performed. As expected, an enhanced number of differentially expressed genes could be detected in alternative versus gold standard DC, with many genes belonging to immunologically relevant pathways. Interestingly, the secretion of a number of pro-inflammatory chemokines was enhanced in alternatively matured DC. Consequently they displayed an enhanced ability to recruit other immune cells to the vaccine DC proximity. Alterations in the set of expressed chemokines might be important for the choice of DC for the treatment of tumors known to be susceptible to a particular effector type attracted by the different chemokines.

**Table 1:** Characteristics of the DC maturation cocktails

Name	Components	Concentrations	
Gold St.	TNFα + IL1β + IL6 + PGE_2_	CL097: 3 μg/mL IFNα: 3,000 U/mL IFNγ: 500 U/mL IL1β: 5 ng/mL IL6: 5 U/mL	MPLA: 4 μg/mL PGE_2_: 1 μM PolyIC: 1 μM TNFα: 100 ng/mL
Alt-1	TNFα + IL1β + IFNα + IFNγ + pIC		
Alt-2	IFNγ + MPLA		
Alt-3	TNFα + IL1β + IFNγ + CL097

**Table 2:** Summary of fast DC characteristics

Molecule	Gold st DC	Alt-1 DC	Alt-2 DC	Alt-3 DC
CD40^a^	3.7 ± 0.2	10.6 ± 0.8	10.6 ± 0.7	8.1 ± 0.6
CD54^a^	3.6 ± 0.1	6.2 ± 0.3	6.9 ± 0.2	3.8 ± 0.2
CD80^a^	2 ± 0.1	4.4 ± 0.2	4.2 ± 0.3	3.5 ± 0.2
CD83^a^	5.8 ± 0.4	4.7 ± 0.4	7.7 ± 0.5	3.0 ± 0.2
CD86^a^	8.6 ± 0.7	8.8 ± 1.0	13.4 ± 1.1	4.2 ± 0.3
IL12^b^	35 ± 15	8,908 ± 1,466	7,283 ± 1,378	2,804 ± 483
IL12^c^	31 ± 9	828 ± 175	583 ± 215	47 ± 10

^a^x-fold in MFI to immature DC; mean ± SE of >10 donors.

^b^pg/mL secreted during the 18 h maturation period; mean ± SE of >10 donors.

^c^pg/mL secreted by mature DC re-seeded at 10^6^ cell/mL for 24 h; mean ± SE of >7 donors.

## Methods

### Chemicals and mAb used

Monophosphoryl lipid A (MPLA) and the imidazoquinoline compound CL097 were purchased from InvivoGen, IL1β from Biosource, IFNα2b from ProSpec, IFNγ from Immunotools, tumor necrosis factor (TNF)α from Peprotech, polyinosinic:polycytidylic acid (polyIC) and prostaglandin (PG)E_2_ from Sigma-Aldrich, whereas IL6 was a kind gift from S. Rose-John (Institute of Biochemistry, University of Kiel, Kiel, Germany).

Following monoclonal antibodies (mAb) were used: PE anti-HLA-DR, Alexa Fluor®700 anti-CD3, eFluor^TM^450 anti-CD16, PE-Cy7 anti-CD14, allophycocyanin-efluor780 anti-CD4 (eBioscience/Affimetrix GmbH), PE-Cy7 anti-CD56 (Beckman Coulter), FITC anti-Vδ2 TCR, allophycocynin anti-CD107a, BV570 anti-CD8α (Biolegend Inc.), allophycocyanin anti-CD14, allophycocyanin anti-CD25, PE anti-CD127, PE-Cy7 anti-CD33, FITC anti-CD11b (Becton–Dickinson) and PE anti-CCR7 (R&D System).

### DC purification, differentiation and maturation

Buffy coats from healthy donors were obtained from the blood bank, whereas samples from melanoma and renal cell carcinoma (RCC) patients were obtained from the Clinic of Dermatology (Wolfgang Marsch) and the Clinic of Urology (Paolo Fornara) of the University Hospital Kroellwitz of the Martin Luther University Halle-Wittenberg in Halle (Saale). Samples were provided upon written informed consent and the procedure was approved by the University ethic committee. Overall, blood samples of more than 20 different healthy donors as well as 4 melanoma and 3 RCC patients were employed in the different experiments.

Peripheral blood mononuclear cells (PBMC) were obtained by gradient centrifugation on a Pancoll gradient (Pan-biotech) using Leucosep tubes (Greiner bio-one). The isolated cells were either frozen in 90% FBS (Life technologies) and 10% DMSO (Carl Roth GmbH) until their use for functional analysis or directly subjected to magnetic purification of CD14^+^ monocytes by mean of CD14-conjugated beads (Miltenyi biotec) following manufacturers’ instructions.

Monocytes were differentiated as previously described [11]. Briefly, 10^6^ monocytes/mL were differentiated in CellGro medium (CellGenix) with 200 ng/mL granulocyte macrophage colony-stimulating factor (GM-CSF; Leukine; Bayer HealthCare) and 1,000 U/mL IL4 (Immunotools,) for 24–36 h and then stimulated for 18 h with the following maturation cocktails (see also Table 1): (1) gold standard (TNFα + IL1β + IL6 + PGE_2_); (2) alternative (alt-)1, or alpha-type1 polarising (TNFα + IL1β + IFNα + IFNγ + polyIC); (3) alt-2 (IFNγ + MPLA) and (4) alt-3 (TNFα + IL1β + IFNγ + CL097). Mature DC were evaluated for phenotype by flow cytometry as previously described [11] and then subjected to mRNA extraction or re-seeded in the absence of further stimuli to evaluate the fate of “wash out” cells. After 24 h, the “wash out” supernatants were collected and kept frozen at −20°C until further use while the cells were lysed with Trizol reagent (Invitrogen) for mRNA extraction.

### cDNA array evaluation of mature DC

The gene expression patterns of immature DC and DC stimulated with gold standard, alt-1 and alt-2 cocktails were compared at the transcriptomic level using the Hs-CCDTM36k oligo array printed at Immunogenetics lab, Department of Transfusion Medicine, Clinical Center, National Institutes of Health. Briefly, 10^6^ cells were lysed in Trizol reagent (Invitrogen) and RNA was isolated following manufacturers’ instructions. 3 µg of total RNA/sample was amplified as previously described [12]. RNA obtained by pooling PBMCs from six normal donors served as a reference and was amplified as the test samples. aRNAs were directly labeled using ULS aRNA Fluorescent Labeling kit (Kreatech) with Cy3 for reference and Cy5 for test samples and co-hybridized to a 36k whole transcriptome human oligo array for 18 h at 45°C before the arrays were washed and scanned by Agilent Scanner (Agilent Biotechnology) [13]. The data were uploaded to the mAdb database at http://nciarray.nci.nih.gov and further analyzed using BRBArray-Tools developed by the Biometric Research Branch, National Cancer Institute (http://linus.nci.nih.gov/BRB-ArrayTools.html) [14]. Class comparison was performed using F test to identify differentially expressed genes among experimental groups and differentially expressed genes were defined as p value <0.005.

### Quantification of chemokines at mRNA and protein level

For validation of the array data quantitative real time PCR (qPCR) was performed. Briefly, 1–2 μg RNA were retro-transcribed into cDNA using RevertAid H minus first strand cDNA synthesis kit (Fisher Scientific) followed by amplification of target genes using the platinum Sybr Green qPCR Supermix (Invitrogen) and a RotorGene6000 machine (Qiagen). The qPCR reaction consisted of 5 min denaturation at 95°C, followed by 40 cycle of 10 s at 72°C and 30 s at 62°C. Melting curve analysis of the qPCR reactions were used to evaluate the specificity of amplification. The transcription was quantified using the ΔCt method and data were normalized to GAPDH and HPRT1 as house-keeping genes. Following primers were used: GAPDH (GAGAAGGCTGGGGCTCATTTGC, GGACTGTGGTCATGAGTCCTTCC), HPRT1 (GCTGGATTACATCAAAGCACTG, CTGACCAAGGAAAGCAAAGTCT), CCL2 (CCCCAGTCACCTGCTGTTAT, TGGAATCCTGAACCCACTTC), CCL3L (CTCTGCAACCAGGTCCTCTC, TTTCTGGACCCACTCCTCAC: amplify CCL3L1 and CCL3L3, but not CCL3), CCL4 (TACCATGAAGCTCTGCGTGA, TACCACAAAGTTGCGAGGAA; amplify CCL4, CLL4L1 and CCL4L2), CCL5 (CGCTGTCATCCTCATTGCTA, ACACACTTGGCGGTTCTTTC), CCL8 (CCCTCCAAGATGAAGGTTTC, GAATCCCTGACCCATCTCTC), CCL13 (TGCACTCAACGTCCCATCTA, ACTTCTCCTTTGGGTCAGCA), CCL17 (TTCAAAACCAGGGTGTCTCC, CTGCCCTGCACAGTTACAAA), CCL22 (ACTGCACTCCTGGTTGTCCT, GCTCTTCATTGGCTCAGCTT), CCL23 (TTTGAAACGAACAGCGAGTG, TGTGTCCCTTCACCTTGACA), CXCL8 (TTGCCAAGGAGTGCTAAAGAA, CAGACAGAGCTCTCTTCCATCA), CXCL9 (TGCTGGTTCTGATTGGAGTG, TTTGGCTGACCTGTTTCTCC), CXCL10 (CAACACGTGGACAAAATTGG, TCCAGTCTCAGCACCATGAA), CXCL11 (AGAGGACGCTGTCTTTGCAT, TAAGCCTTGCTTGCTTCGAT), CXCL16 (CTCCTGGCCATCATCTTCAT, AAGCTTCCATTCTTGGCTCA),

The chemokine concentrations in the DC “wash out” supernatants were determined by sandwich ELISA for CCL2, CCL3, CCL5, CCL8, CXCL9 and CXCL16 (all from PeproTech) and by flow cytometric plexing for CCL4/MIP-1β, CXCL8/IL8 and CXCL10/IP-10 (CBA multiplexing kit, Becton–Dickinson). Expression of transmembrane CXCL16 on mature DC was evaluated by flow cytometry using a biotinylated anti-CXCL16 Ab (PeproTech) followed by streptavidin PE (eBioscience/Affimetrix).

### DC migration assay

Mature DC (2 × 10^4^) were seeded in 200 μL medium in a 4 μm thincert insert well (Greiner bio-one), while in the lower chamber of the 24 well plate 600 μL medium containing 100 ng/mL of CCL21 (Peptotech) were added. After 3 h incubation at 37°C, cells migrated into the lower compartment were collected. Samples were analysed on a Navios (Beckman Coulter) flow cytometer under constant flow for 2 min after addition of propidium iodide in order to discriminate dead cells. Counting beads (Beckman Coulter) were also added to the samples directly before measurement in order to normalise acquisition rates among the samples.

### PBMC migration assay

**Figure 1:**
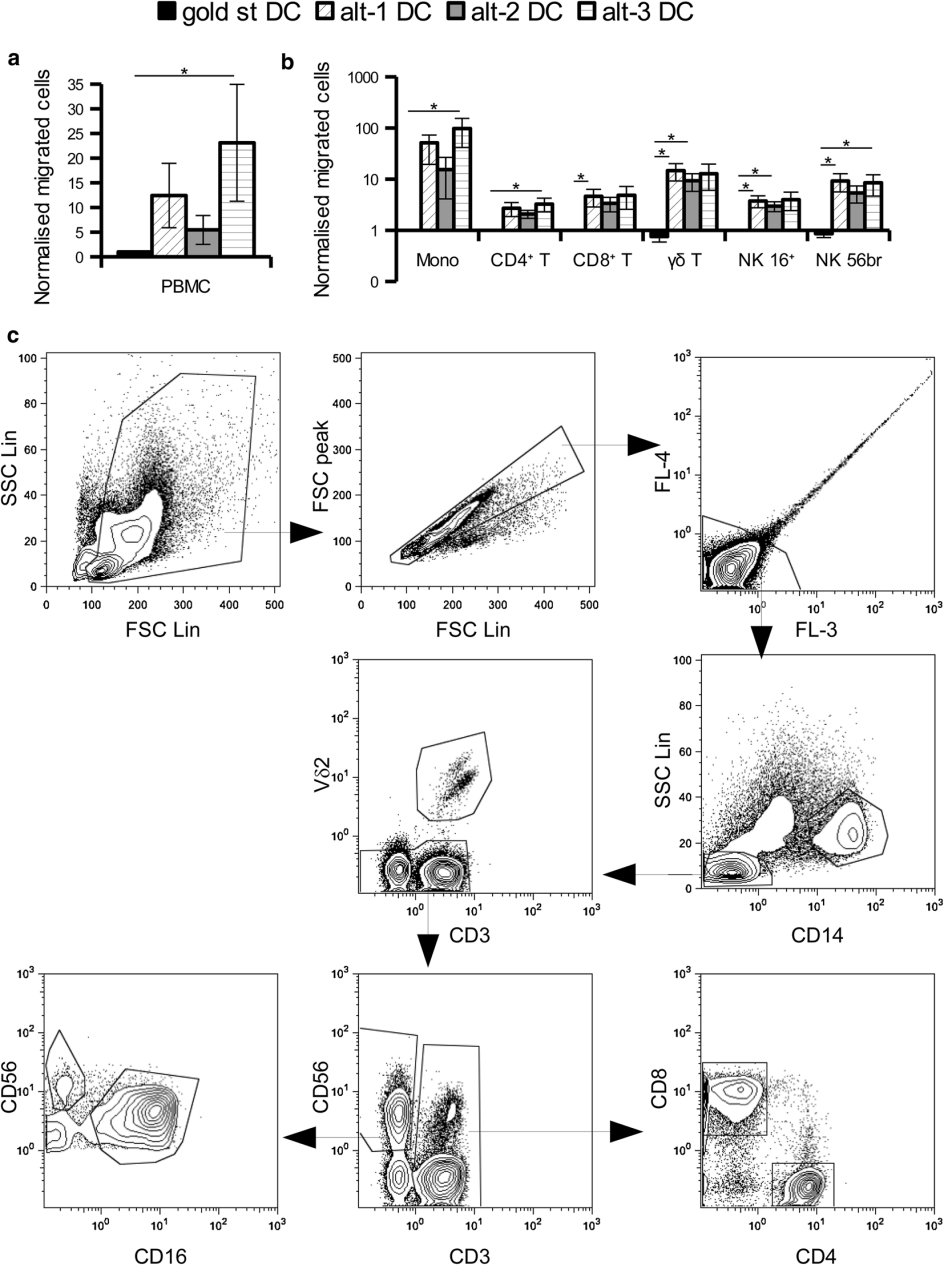
Enhanced migration of healthy donor PBMC toward conditioned supernatants from alternative DC. “Wash out” supernatants collected 24 h after seeding mature DC in the absence of exogenous stimuli were used as a source of chemoattractants for immune cells. Total PBMC from healthy donors were loaded in a trans-well insert (3 μm pores) and allowed to migrate toward medium or DC “wash out” supernatants for 3 h. Migrated cells were analysed by flow cytometry to determine their total number (**a**) as well as their distribution into the different immune cell subpopulations (**b**) by using the indicated gating strategy (**c**). Data from seven different experiments are reported as mean ± SE after normalization to supernatants from gold standard DC. *p < 0.05 in the Tukey post hoc test.

**Figure 2:**
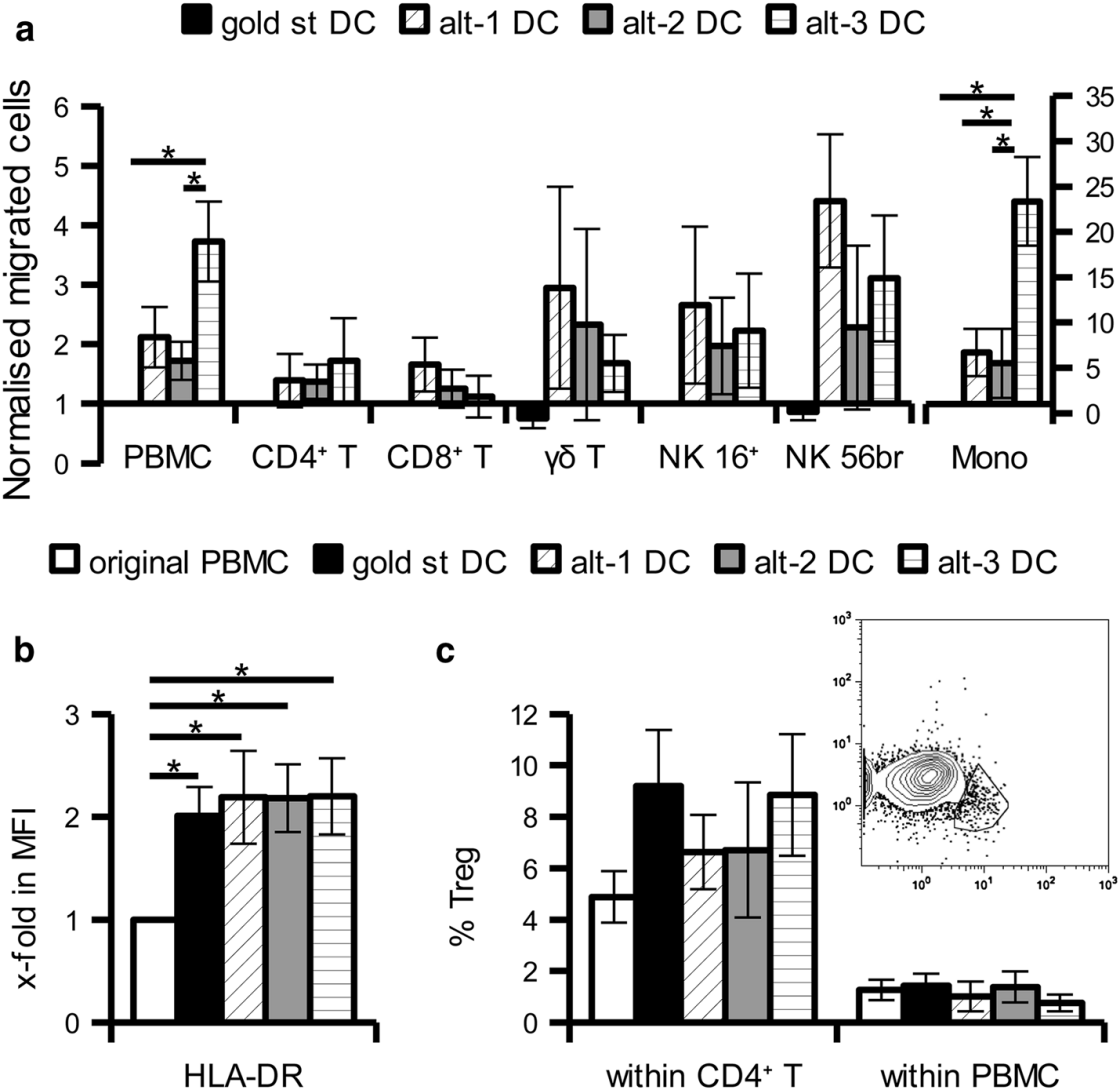
Effects of DC conditioned supernatants on PBMC from cancer patients. Total PBMC from melanoma or RCC patients were added into trans-well insert (3 μm pores) and allowed to migrate toward DC “wash out” supernatants for 5 h. Migrated cells were evaluated by flow cytometry to determine absolute number and immune composition. **a** Mean ± SE of the x-fold increase in the migration of the different immune populations over gold standard DC-derived supernatants from 4 to 6 different experiments is shown. **b** x-fold increase in the HLA-DR MFI of migrated CD14^+^ monocytes versus the original population. **c** Gating strategy (insert) and percentage of CD25^high^ CD127^low^ Treg of the original PBMC and among migrated cells shown as percentage of CD4^+^ T cells or of total leukocytes. *p < 0.05 in the Tukey post hoc test.

Cryopreserved total PBMC from healthy donors and cancer patients were thawed and 1.5 × 10^6^ cells were seeded in 300 μL medium in a 3 μm Thincert insert well (Greiner bio-one). In the bottom chamber of the 12 well plate 1 mL of medium or different dilutions (1:2 to 1:3) of the “wash out” supernatants from the different DC were added as chemoattractant. After 3 h incubation at 37°C, cells from the bottom chamber were collected and stained with fluorochrome-labelled antibodies in order to identify the different immune populations, namely monocytes (as CD14^+^, SSC^high^), CD4^+^ and CD8^+^ T cells (as CD3^+^ and CD4^+^ or CD8^+^, respectively), γδ T cells (as CD3^+^ Vδ2 TCR^+^) and the two natural killer (NK) subpopulations of CD56^br^ and CD16^+^ cells (as CD3^neg^ and CD56^br^/CD16^neg^ and CD56^dim^/CD16^+^, respectively). PBMC from cancer patients were also evaluated for the phenotype of monocytes by evaluating expression of CD33, CD11b and HLA-DR and for the presence of regulatory T cells (Treg) as CD25^high^ CD127^low^ CD4^+^ T cells. Examples of gating strategy are given in Figures 1c and 2c.

Samples were measured on a Navios (Beckman Coulter) flow cytometer under constant flow for 2 min after addition of propidium iodide in order to discriminate dead cells. Counting beads (Beckman Coulter) were also added to the samples directly before acquisition to further normalise acquisition rates among samples.

### Functional evaluation of migrated cells

Mature DC (5 × 10^5^) were seeded in 1 mL in the lower chamber of a 12 well plate. After 4 h preincubation 2.5–3 × 10^6^ thawed autologous PBMC were added in a 3 μm Thincert insert well and allowed to migrate. After 18 h the migrated cells were collected and functionally evaluated as previously described [11]. Briefly, the ability to secrete IFNγ was determined using a secretion assay kit (Milteny biotech) according to the manufacturer’s instruction, whereas their cytotoxic activity was analysed employing the CD107a degranulation assay after 4 h incubation with 10^5^ Daudi cells. For evaluation of T cell functionality the mature DC were pulsed with 1 μg of SEB for 30 min at 37°C. After washing and 4 h pre-incubation for chemokine secretion, PBMC were added and allowed to migrate for 3–4 h before undergoing the IFNγ secretion assay.

### Statistical analysis

Differences between treatments were evaluated using the one-way ANOVA test for correlated samples and the Tukey post hoc test. Values of p < 0.05 were considered significant.

## Results

### Increased alteration of the DC transcriptome using alternative maturation cocktails

**Figure 3:**
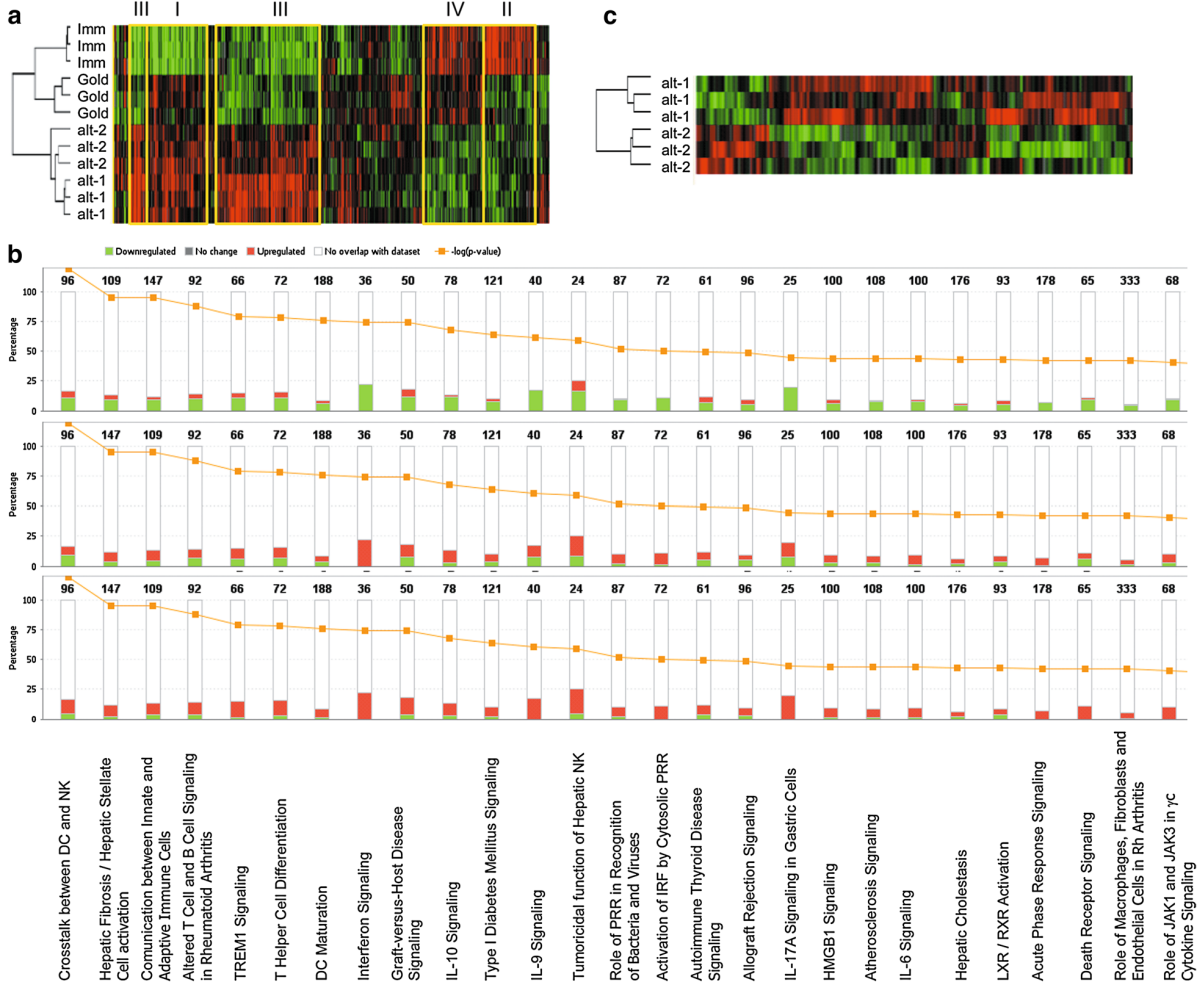
Effect of different maturation cocktails on DC transcriptome. Immature DC were compared to cells stimulated with gold standard, alt-1 and alt-2 maturation cocktails using cDNA array. Three different donors were used for the evaluation. The heat map of genes differentially regulated among the four DC types (**a**) or just the two alternative DC (**c**) are shown. **b** Distribution of the differentially expressed genes in the canonical pathways is shown for each mature DC (gold standard DC, *top;* alt-1 DC, *middle*; alt-2 DC, *bottom*) versus the immature DC. The *numbers above the bars* indicate the number of genes belonging to the different pathways, whereas the *yellow line* indicates the -Log10 of the p value of the modulation for each family.

We previously characterized the phenotype and functional activity of monocytes differentiated into DC using the shortened “FastDC” protocol and stimulated with the clinical gold standard cytokine cocktail or with alternative cocktails based on a combination of different TLR ligands with IFNγ (for detailed composition see Table 1). Now, a transcriptome-wide comparison was performed by cDNA array using immature, gold standard and the alt-1 and alt-2 cocktails, previously demonstrated to have the highest functional interaction with effector cells [11]. Comparison of immature DC with DC stimulated with the three cocktails resulted in a significant altered expression pattern with 483 differentially expressed genes reaching statistical significance by F test (p < 0.005; Figure 3a). Among them, 78 genes were up-regulated (highlighted as box I in Figure 3a) and 86 down-regulated (box II in Figure 3a) in all three mature DC versus immature DC, whereas the two alternatively matured DC had additional 172 genes up-regulated and 80 down-regulated genes when compared to both immature and gold standard DC (Figure 3a, box III and IV, respectively). Evaluation of the classes and canonical pathways of differentially expressed genes highlighted the highest significance in changes of genes involved in the cross-talk of DC and innate cells, in T helper differentiation and in IFN signalling (Figure 3b). Further comparison between alt-1 and alt-2 DC revealed 244 significantly differentially expressed gene with 181 up-regulated in the alt-1 and 63 in the alt-2 DC (Figure 3c). Analysis of the canonical pathway demonstrated a reduced expression of genes associated with cell death and an enhanced expression of pathways associated with cell movement, immune cell trafficking and inflammation response in alt-2 DC versus alt-1 DC (data not shown).

### Influence of maturation cocktails on the chemokine expression in DC

A high number of genes identified as differentially expressed in the various DC belong to the chemokine family. Since chemokines are known to play a key role in the induction of immune responses and homing of immune effector cells, the validation of the array data was focused on these molecules. Two expression patterns could be distinguished among the differently matured DC from the array data: CCL17, CCL22 and CXCL16 were predominantly up-regulated in gold standard DC, while the two alternative DC had an enhanced expression of CCL3-like3 (CCL3L3), CCL4-like1 (CCL4L1) and CCL4L2, CCL5, CXCL8, CXCL9 and CXCL11 (Table 3).

**Table 3:** cDNA array data of chemokine and chemokine receptor genes

Array rank	Gene	Univariate F test p value	Geometric mean of ratios				Pairwise significant (p < 0.01)^a^
			Imm (#1)^a^	Gold st (#2)^a^	Alt-1 (#3)^a^	Alt-2 (#4)^a^	
2	CCL5	1.00E−007	0.04	0.06	3.18	2.53	(1.3) (1.4) (2.3) (2.4)
16	CCL17	2.90E−006	2.29	63.78	31.39	47.56	(1.2) (1.3) (1.4)
18	CCL4L1	3.40E−006	0.46	0.57	3.47	2.44	(1.3) (1.4) (2.3) (2.4)
28	CCL4L2	7.10E−006	1	1.07	11.43	7.7	(1.3) (1.4) (2.3) (2.4)
167	CXCL8	4.98E−004	0.02	0.1	0.44	0.32	(1.2) (1.3) (1.4) (2.3)
195	CCL3L3	7.85E−004	6.58	1.9	6.61	3.79	(2.1) (2.3) (2.4)
247	CCR7	1.32E−003	0.85	4.74	12.02	8.41	(1.2) (1.3) (1.4)
257	CXCL11	1.41E−003	0.4	0.4	1.15	2.63	(1.4) (2.4)
391	CXCL9	3.03E−003	0.55	0.44	8.75	10.61	(1.3) (1.4) (2.3) (2.4)
411	CCL22	3.44E−003	NA^b^	47.6	11.42	31.93	
458	CXCL16	4.35E−003	0.79	1.79	1.01	0.93	(1.2) (3.2) (4.2)

^a^DC code for statistical comparison.

^b^Value not available.

**Figure 4:**
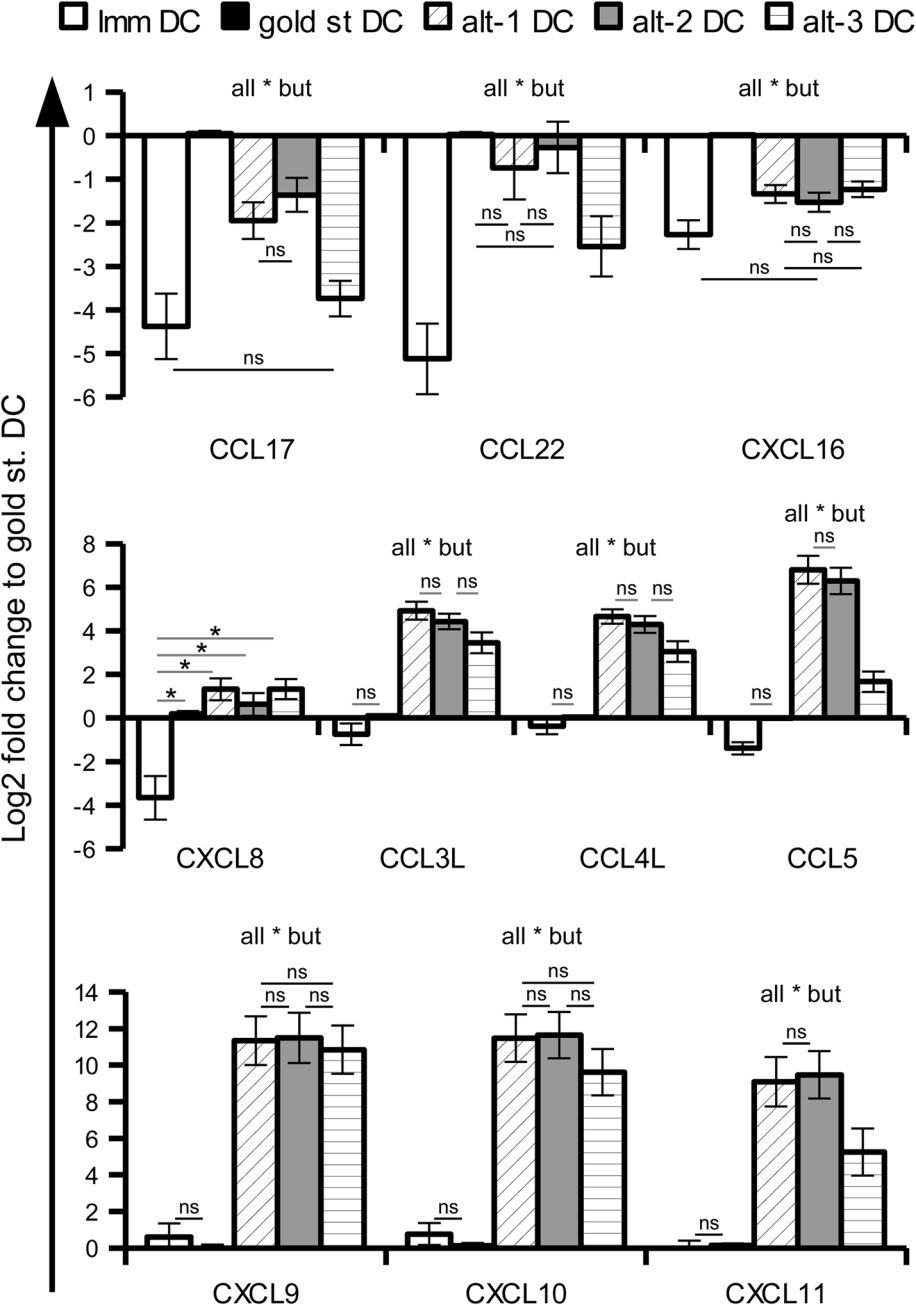
qPCR evaluation of chemokine gene expression in differently matured DC. The expression levels of chemokine genes in immature DC as well as in DC stimulated with gold standard, alt-1, alt-2 and alt-3 maturation cocktails were evaluated by qPCR. Results were first normalised to the mean expression of two housekeeping genes (GAPDH and HPRT1) and then toward the gold standard DC. The results are represented as the mean ± SE of 9–12 different donors. *p < 0.05, ns p > 0.05 in the Tukey post hoc test.

Validation of the gene expression pattern of gold standard, alt-1 and alt-2 DC was performed by quantitative real time PCR (qPCR) using healthy donor preparations and confirmed for all chemokines the altered expression pattern highlighted by the mRNA array data (Figure 4). The qPCR analysis of these chemokines was also expanded to DC stimulated with a third alternative cocktail that was not included in the array evaluation due to its slightly lower functionality [11]. The alt-3 DC demonstrated lower expression levels of CCL3, CCL4, CCL5 as well as of CXCL11 when compared to alt-1 and alt-2 DC (Figure 4). Furthermore, the expression of CXCL10, representing in addition to CXCL9 and CXCL11 a ligand of CXCR3, was highly up-regulated in alt-1 and alt-2 DC, but slightly less and statistically not significant in alt-3 DC (Figure 4).

**Figure 5:**
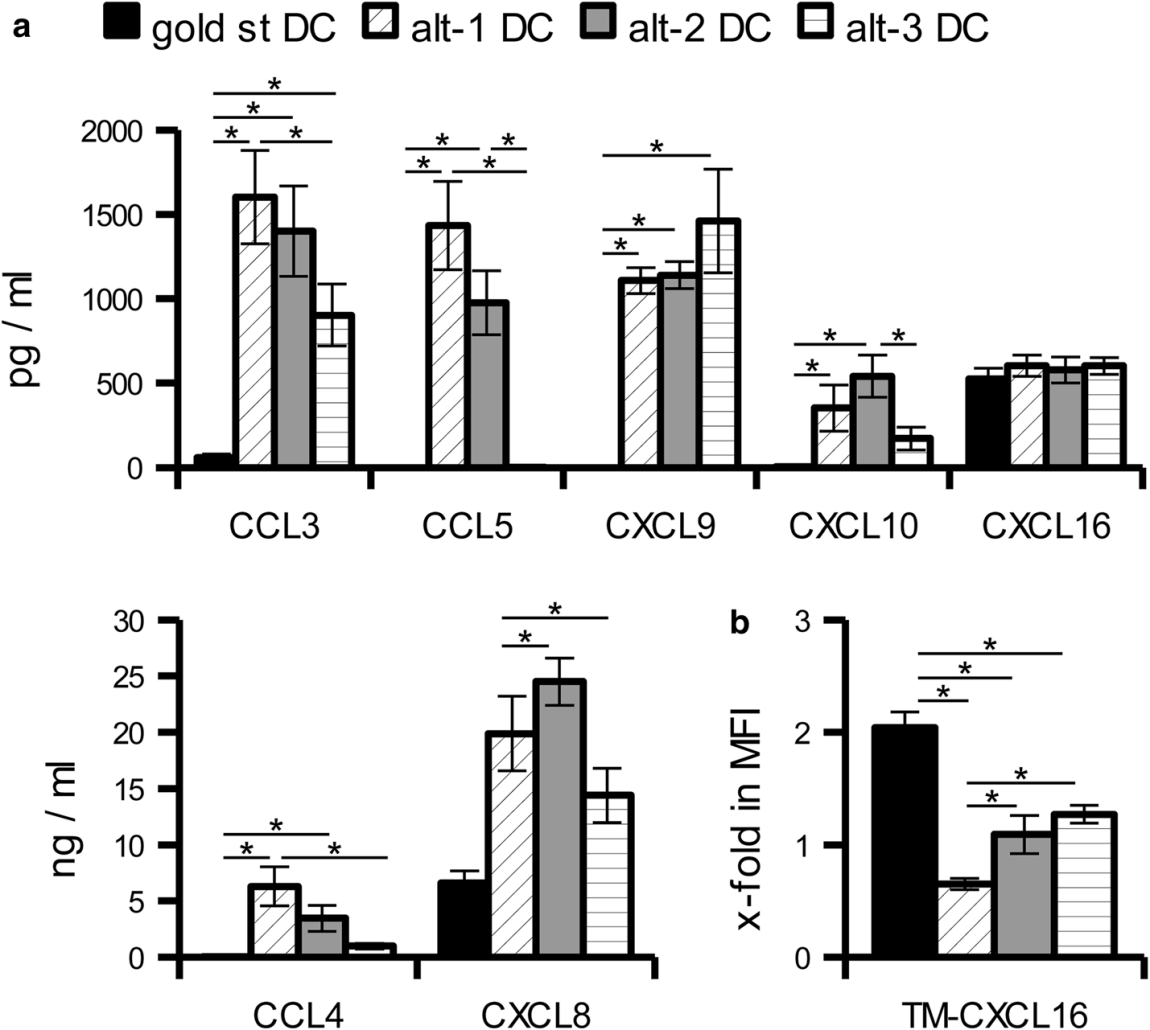
Evaluation of chemokine production at the protein level in differently matured DC. **a** “Wash out” supernatants collected 24 h after seeding mature DC in the absence of exogenous stimuli were used to evaluate chemokine secretion by sandwich ELISA or flow cytometry based plexing. The results are represented as the mean ± SE of 4–7 different donors. **b** The presence of transmembrane (TM)-CXCL16 on mature DC was evaluated by flow cytometry. The results are shown as mean ± SE of the x-fold increase in MFI over immature DC determined from ten different donors. *p < 0.05 in the Tukey post hoc test.

The differential expression pattern of selected chemokines was also validated at the protein level using supernatants obtained by re-seeding the mature DC in the absence of further stimuli (“wash out” supernatant) for 24 h, a situation mimicking the secretion after in vivo injection. In line with the mRNA data, gold standard DC secreted marginal amounts of CCL3, CCL4, CCL5, CXCL9 and CXCL10 in contrast to alt-1 and alt-2 DC. Alt-3 DC shared high levels of CXCL9 secretion with alt-1 and alt-2 DC, but did not secrete CCL5 and produced much lower levels of CXCL10, CCL3 and CCL4. CXCL8 was secreted by all mature DC, but always at higher levels in the alternatively matured DC. Despite the differences at the mRNA level CXCL16 was present at comparable levels in the supernatants of the different DC (Figure 5a). Since CXCL16 is also a transmembrane molecule flow cytometry was performed on mature DC demonstrating a surface staining for CXCL16 on gold standard, but not on alternative DC (Figure 5b).

### Long lasting production of chemokines

**Figure 6:**
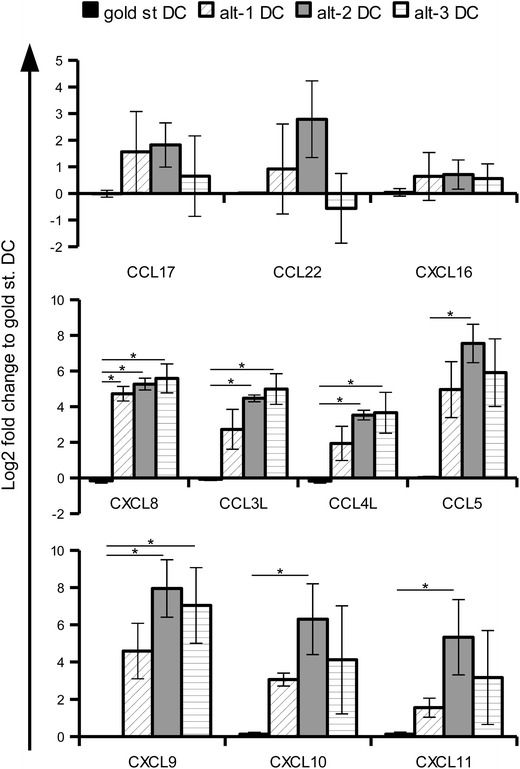
qPCR evaluation of chemokine gene expression in 24 h “wash out” DC. Mature DC were seeded for 24 h in the absence of exogenous stimuli and then collected for mRNA extraction and qPCR evaluation. Results were first normalised to the mean expression of two housekeeping genes (GAPDH and HPRT1) and then toward the gold standard DC. The results are represented as the mean ± SE of three different donors. *p < 0.05 in the Tukey post hoc test.

In order to further characterise the chemokine secreting capabilities of the different DC upon in vivo application, transcriptional profile of the wash out DC was evaluated by qPCR. Even 24 h after maturation, the alternative DC were still transcribing higher levels of CCL3L, CCL4L, CCL5, CXCL8, CXCL9, CXCL10 and CXCL11 in comparison to the gold standard DC (Figure 6). CCL17 and CCL22, that were prevalently expressed in the mature gold standard DC, displayed a more heterogeneous expression among the different donors in the wash out DC, resulting in no statistically significant difference among the various maturation cocktails.

### Chemokine receptor expression by the differently matured DC

**Figure 7:**
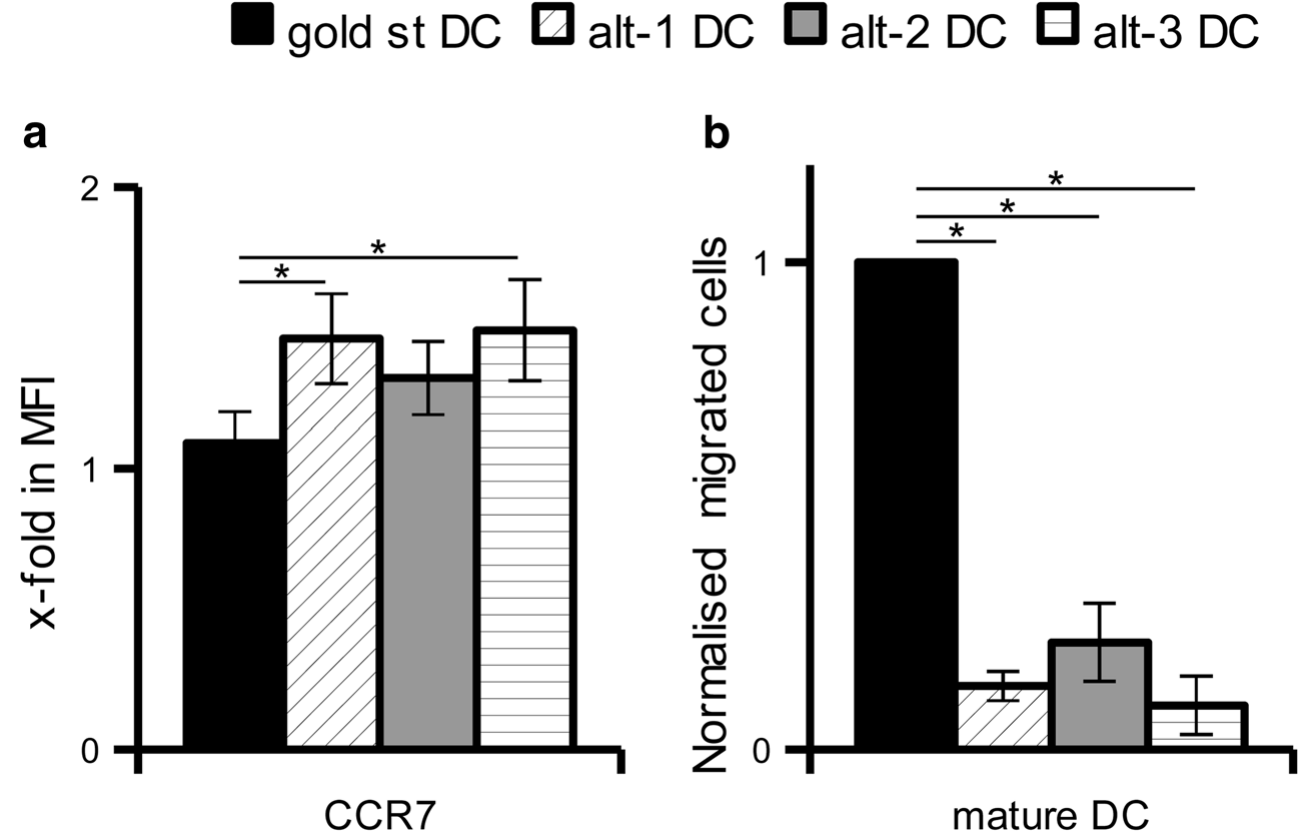
Expression and functionality of CCR7 by differently matured DC. **a** Mature DC were evaluated by flow cytometry for the expression of CCR7. The x-fold increase in MFI over immature DC is show as mean ± SE of 13 different donors. **b** The ability of mature DC to migrate toward the CCR7 ligands CCL21 was evaluated in a trans-well migration assay. After 3 h incubation at 37°C, DC migrated in the lower chamber were counted by flow cytometry. The results of three experiments are represented as mean ± SE upon normalization to the gold standard DC. *p < 0.05 in the Tukey post hoc test.

In addition to the various chemokine genes, one chemokine receptor, namely CCR7 was also found to be significantly upregulated in all mature DC (Table 3). Flow cytometric analysis confirmed the enhanced expression of this receptor on all mature DC, with the highest expression levels in alt-1 and alt-3 DC (Figure 7a). In contrast, functional analysis of the receptor determined by a migration assay toward CCL21 demonstrated that gold standard DC possess a higher migratory capacity (Figure 7b). These data confirm previously published results describing a missing correlation between CCR7 expression levels and functionality [15–17].

### Chemoattractive properties of “wash out” DC supernatants

The functional relevance of the chemokine secretion by the various DC was then evaluated in a classical trans-well migration assay using total PBMC from healthy donors and the “wash out” supernatants. After 3 h incubation, only few cells had actively migrated towards the gold standard DC. In contrast, the supernatants from the alternatively matured DC attracted a 5- to 25-fold higher number of immune cells (Figure 1a).

Immunophenotyping of the migrated cells revealed that most of the cells migrating toward the alternative DC supernatants were CD14^+^ monocytes, with alt-3 DC providing the highest and alt-2 DC the lowest rate of migration among the alternative DC. In addition effector cells from the innate and adaptive immunity were attracted by the alternative DC, but to a much lower extent than monocytes (Figure 1b).

### Evaluation of monocyte attracting chemokines

**Figure 8:**
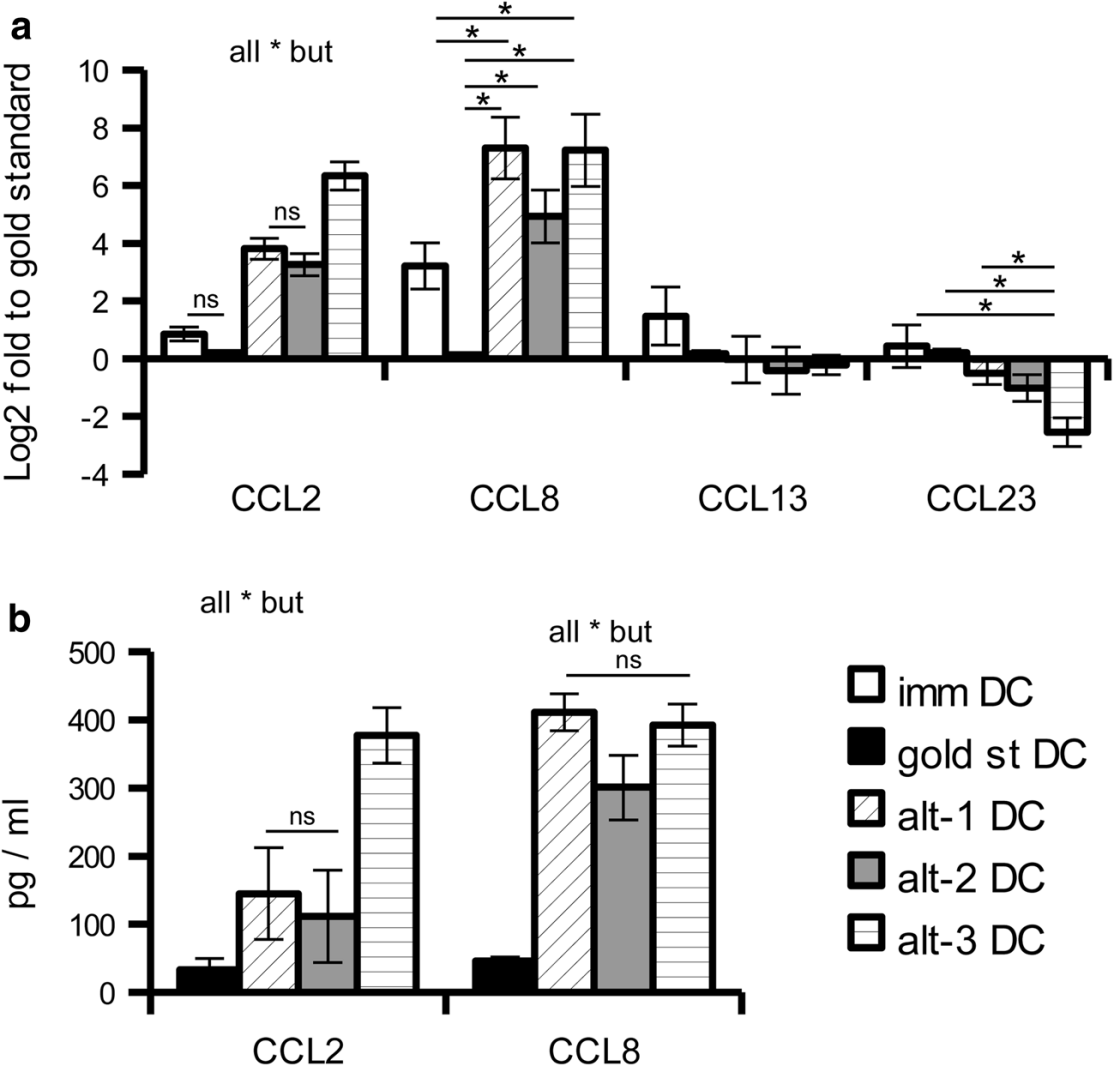
Determination of chemokines involved in monocyte chemoattraction. **a** The expression of different members of the monocyte chemoattractant protein family was evaluated by qPCR. Results are first normalised to the mean expression of two housekeeping genes (GAPDH and HPRT1) and then toward gold standard DC. The relative expression of nine different donor is represented as mean ± SE. *p < 0.05, ns p > 0.05 in the Tukey post hoc test. **b** The CCL2 and CCL8 concentration of “wash out” supernatants from mature DC seeded in the absence of exogenous stimuli for 24 h were determined by sandwich ELISA. The results are represented as mean ± SE of 8–12 different donors. *p < 0.05, ns p > 0.05 in the Tukey post hoc test.

Due to the increased attraction of monocytes by alt-3 DC that were not present in the array evaluation, the transcription of chemokines involved in monocyte attraction (i.e. CCL2, CCL8, CCL13 and CCL23) was determined in the various DC by qPCR. Among the genes analysed, only CCL2 was specifically up-regulated in alt-3 DC, with a more than 100- or 10-fold increase in transcription when compared to immature and gold standard or alternative DC, respectively. CCL8 was also up-regulated in alt-3 DC, but the expression was shared by alt-1 DC (Figure 8a). These data were further confirmed at the protein level in “wash out” supernatants (Figure 8b).

### Functionality of migrated effector cells

To evaluate the functional consequences of the attraction of effector cells, the migration experiments were performed using slightly changed conditions. Instead of “wash out” supernatants, mature DC were seeded in the bottom chamber and pre-cultured for 4 h in order to enrich the culture medium with secreted chemokines. Moreover, to obtain enough number of innate cells for functional evaluation the migration assay was prolonged to 18 h. Finally, for the determination of antigen-specific T cell activation the mature DC were pulsed with the model antigen staphylococcal enterotoxin B (SEB) before seeding.

**Figure 9:**
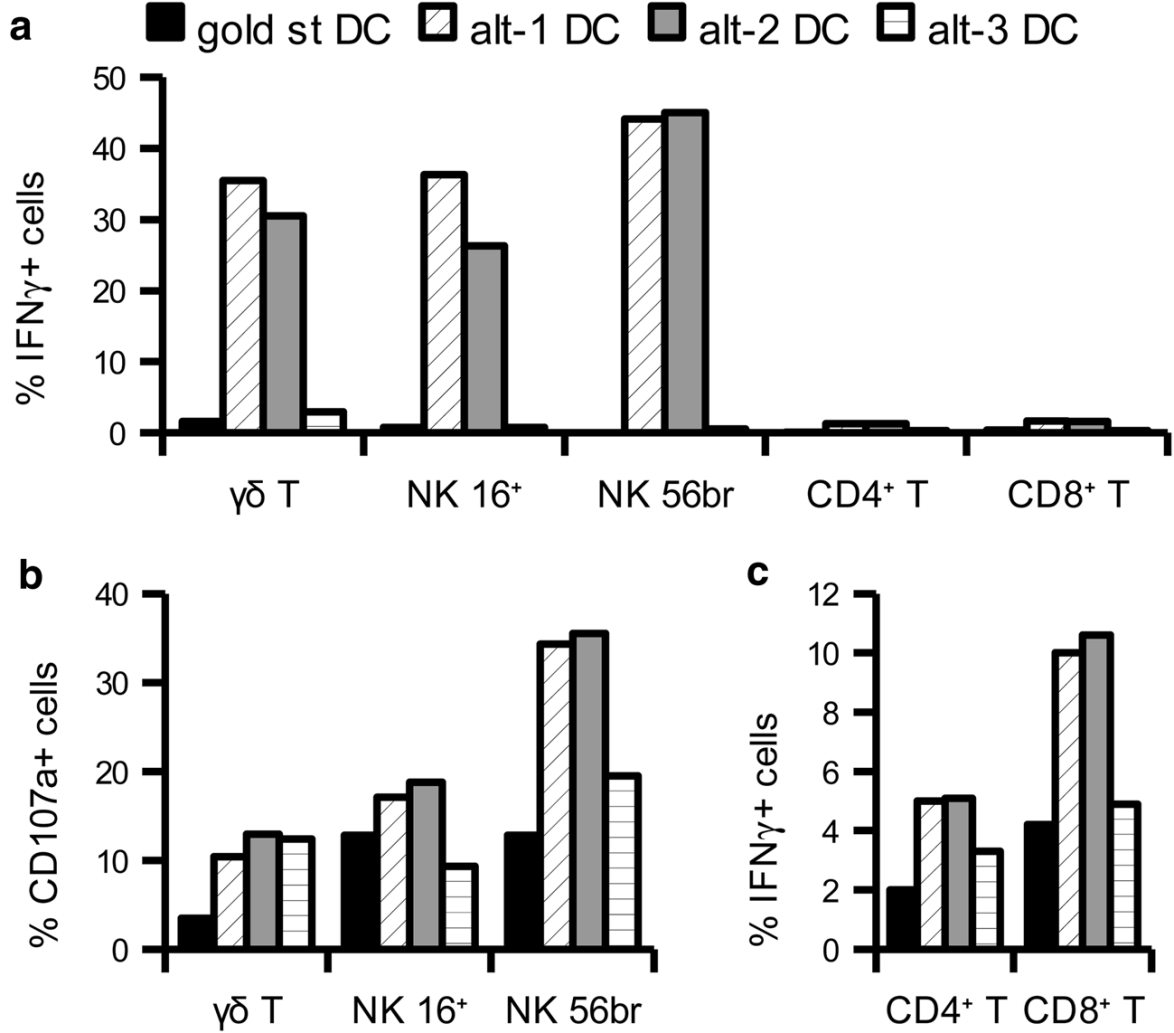
Functional activation of lymphocytes migrated to alternatively matured DC. **a**, **b** 5 × 10^5^ mature DC were seeded in 12 well plate for 4 h, after which 3 × 10^6^ autologous PBMC were added above a 3 μm insert. After 18 h incubation the cells that had migrated into the bottom chamber were taken and either immediately evaluated for IFNγ secretion (**a**) or incubated for 4 h in the presence of Daudi cells and evaluated for CD107a degranulation (**b**). **c** 5 × 10^5^ mature DC were pulsed with SEB for 30 min at 37°C, washed and then seeded in 12 well plate for 4 h, after which 3 × 10^6^ autologous PBMC were added above a 3 μm trans-well insert. After 4 h incubation the cells in the bottom chamber were taken and evaluated for IFNγ secretion. Shown is one representative experiment out of two (**c**) or three (**a**, **b**) with similar results.

In line with the results obtained upon co-culture conditions [11] only γδ T cells and NK cells that had migrated to alt-1 or alt-2 DC were able to secrete IFNγ, whereas no secretion was obtained in response to gold standard or alt-3 DC (Figure 9a). Regarding the acquisition of cytotoxic activity, all innate effector types displayed a basal level of CD107a degranulation in response to further incubation with Daudi cells, but again higher levels were found in response to the alternative, and particularly alt-1 and alt-2 DC (Figure 9b). Shifting to the adaptive immunity, CD4^+^ and CD8^+^ T cells migrating to SEB-pulsed DC were able to secrete IFNγ in an antigen-specific way, which was higher upon interaction with alt-1 and alt-2 DC (Figure 9c).

### Attraction of PBMC derived from cancer patients

“Wash out” supernatants of the differently matured DC were also used to determine the attraction of PBMC derived from patients with melanoma or renal cell carcinoma (RCC) in order to evaluate the behaviour of cells from cancer patients before clinical translation. As shown in Figure 2a an enhanced migration of cells and particularly monocytes was found in response to the “wash out” supernatants of alternative DC, which is in line with the results obtained using PBMC from healthy donors. Phenotypic characterisation of the migrated monocytes revealed an enrichment of cells with high HLA-DR expression levels (Figure 2b). The migrated monocytes displayed also slightly reduced expression of CD11b and CD33 (data not shown) suggesting the recruitment of inflammatory cells and not of CD33^high^ CD11b^high^ HLA-DR^low^ myeloid derived suppressor cells. In addition, comparable and low absolute numbers of CD3^+^ CD4^+^ CD25^high^ CD127^low^ Treg were attracted by the different supernatants. Despite a trend toward enrichment of Treg within the CD4^+^ T cell compartment in response to gold standard and alt-3 DC supernatants was found, it did not reach statistical significance. Moreover, determination of the number of Treg within the total migrated population revealed no overall enrichment in Treg in response to any DC supernatant (Figure 2c).

## Discussion

Many attempts have been performed in order to improve the clinical outcome of cancer patients treated with DC-based immunotherapy. To this purpose continuous optimization of the protocols for the production of vaccine DC are being tested, with changes in the stimuli used for the ex vivo differentiation of CD14^+^ monocytes or CD34^+^ precursor cells as well as for their maturation.

The omic-based technologies now allow not only the comparison of these populations by functional assay, but also at the transcriptomic and proteomic levels. In this way multiple differences between protocols can be identified and their impact on the overall functionality of the vaccine further evaluated.

In this study we focused our attention on the Fast protocol for DC differentiation [5], whose shorter ex vivo cultivation time might support their clinical translation when compared to the 6 day protocol of classical monocyte-derived DC both for reduced costs and decreased possibility of contamination of the vaccine during handling. Regarding the maturation stimuli, the clinical gold standard cytokine cocktail [18] was compared to TLR ligand and IFNγ-containing cocktails, namely the alt-1 or α-type 1 polarising [7] and the alt-2 [8], that have already been characterized at the functional level in a previous report [11].

A larger number of genes were differentially expressed in response to the two alternative cocktails over immature DC than in response to the gold standard, underlining the higher level of differentiation/maturation obtained with such alternative cocktails. The up-regulation of many genes involved in the interaction with innate cells is in line with our previous demonstration of the enhanced ability of such DC to stimulate the cytolytic and cytokine-secreting ability of NK and γδ T cells [11]. Surprisingly, the IL12p35 gene responsible for the formation of the bioactive IL12p70 involved in their activation [11] was not found as significantly regulated by the array analysis.

Different members of the chemokine family were included among the genes with the strongest levels and statistical significance of modulation. Comparison of the differently matured DC highlighted an almost opposite up-regulation of these members between gold standard and alternative DC.

In line with previous studies, stimulation with PGE_2_ inhibited the expression of inflammatory chemokines [19, 20], while inducing the production of CCL17 and CCL22 [21]. Despite the involvement of such chemokines in the attraction of Th2 and Treg cells [22], no statistically significant enhanced migration of Treg toward supernatants from gold standard DC was detected. In addition to the high inter-donor variability in the migration assay on both healthy DC and patient-derived PBMC, a possible explanation might be the concomitant expression of CCL23 (Figure 8a) that has been proposed to contrast Treg recruitment [23].

Another chemokine specifically up-regulated by the gold standard DC is CXCL16 that despite present at comparable levels in the culture supernatants of all mature DC was specifically retained on the surface of gold standard DC. This might be due to the absence in the maturation cocktail of IFNγ that has been reported to foster the shedding of the transmembrane CXCL16 [24]. The functional consequences of such specific CXCL16 retention on gold standard DC is currently under evaluation. Indeed, the specific receptor CXCR6/Bonzo is expressed by CD45RO^+^ memory T cells [25] and has been reported to play an important role for antigen-specific T cell activation [26]. Concerning the innate immunity CXCR6 and CXCL16 play a key role within the liver, where they are involved in the action of NKT cells, either for immunosurveillance [27] or suppression [28], and for hapten-specific memory NK cells [29].

With the exception of CCL17, CCL22 and CXCL16 the gold standard DC were poor producers of chemokines and indeed in functional assay their “wash out” supernatants displayed a reduced ability to induce leukocytes recruitment. In contrast, the alternative DC did up-regulate many inflammatory chemokines belonging to both the CC and CXC family, resulting in enhanced attraction of effector cells in vitro. The retained high expression levels of the encoding mRNA 24 h after the end of the maturation suggest that upon in vivo injection the DC should still be able to produce chemokine and thus facilitate their interaction with effector cells. Whereas alt-1 and alt-2 DC displayed few differences, alt-3 DC obtained by stimulation with a third alternative cocktail [9, 11] presented a slightly different expression pattern. As determined by ELISA or multiplexing, alt-3 DC had a much lower or even absent secretion of CCL3, CCL4, CCL5 as well as of CXCL10 and CXCL11, chemokines for which additional “co-stimulatory” activity on immune cells other than chemoattraction have been reported. These chemokines can induce changes in cell adhesiveness and promote the formation and the stability of the immunological synapse between DC and T cells [30, 31]. Moreover, they have been involved in the promotion of the cytotoxic activity [32–37] and proliferation of NK and T cells [38, 39] and to provide co-stimulatory signals for T cells [40]. Since for the three CXCR3 ligands CXCL9 CXCL10 and CXCL11 different and sometimes opposing function have been reported depending on the particular infection or pathology investigated [41], the different pattern of chemokine secretion among the alternative DC might promote their individual implementation in specific vaccine formulations depending on the particular clinical setting.

Another difference among the various alternative DC was the increased attraction of monocytes by alt-3 when compared to alt-1 and even more to alt-2 DC, an ability that could be linked to their secretion of CCL2 and CCL8. In cancer patients, the enhanced attraction of monocytes was not associated with the phenotype of myeloid derived suppressor cells that could potentially down regulate the vaccine functionality, since there was an enrichment of HLA-DR^high^ migrating monocytes. In light of the demonstration in various murine models that vaccination with DC can also relay on endogenous APC for their immunological functions [42–45], it will be important to evaluate the functional capability of those cancer-associated myeloid cells in order to confirm a possible role in fostering vaccine efficacy. Recently, CCL2 has also been involved in the recruitment of murine γδ T cells as well as human Vδ1^+^, but not of Vδ2^+^ cells [46]. Due to the low amount of Vδ1^+^ T cells in the peripheral blood, an enhanced attraction of this population by alt-3 DC could not be detected. However, this possible interaction might be worth to investigate since different tumor types are infiltrated by Vδ1^+^ T cells [47, 48] and anti-tumor activity against solid as well as hematologic malignancies have been reported both in vitro [49] and in xenograft transplantation models [50, 51], resulting in the establishment of expansion protocol for clinical therapy [52].

Opposite to the enhanced chemo-attracting capacity of the alternative DC are their reduced migratory properties. Indeed, despite an upregulation of CCR7 even higher than gold standard DC, alternative DC displayed a reduced migration in vitro toward its ligand CCL21 and would then have poor migration to the lymph node upon subcutaneous or intradermal injection into cancer patients. Two possible approaches can be used to overcome this problem. On one side, further in vitro manipulation can be performed to enhance the receptor functionality. Pretreatment of murine DC with recombinant CCL17 or CCL22 has been demonstrated to enhance their responsiveness to CCR7 as well as CXCR4 ligands without affecting the receptor expression nor being dependent on CCR4 expression [53]. Similarly, addition of LTC_4_ to various maturation stimuli was able to enhance CCR7 responsiveness to its ligands with a similar potency to PGE_2_ but without impairing the DC capability to secrete functional IL12 [54]. Otherwise the in vivo injection route can be modified shifting from the subcutaneous or intradermal route to the usage of intralymphatic cannulation or direct intranodal injection. Indeed, despite some clinical trials directly comparing the different routes of injection have highlighted better results with the former two sites, they were also performed with a “not yet optimised” DC formulation, in which the in vivo migration step would still be required to further select the most mature DC within the vaccine [55–58]. Production of DC vaccine with new formulations like the proposed alternative cocktails that lead to a highly homogeneous and mature DC population would not need further in vivo selection and could thus be directly delivered into the lymph nodes to attract effector cells and induce their functional activation bypassing their poor migratory capacity.

Overall, this study demonstrated a higher functionality of alt-1 and alt-2 DC over gold standard DC. Despite sharing many differentially expressed genes, the two alternative DC displayed also some differences that need to be evaluated in order to determine the consequence on the activity of the DC vaccine. Furthermore, a reduced chemoattractant property of gold standard DC was identified, which might represent a possible cause in addition to the missing IL12p70 secretion for their poor clinical efficacy.

## Conclusions

In this study we demonstrated that vaccine DC stimulated with the gold standard maturation cocktails display a limited production of chemokines resulting in a reduced ability to recruit effector cells in their proximity. In contrast, stimulation of DC with cocktails based on IFNγ and different TLR ligands resulted in enhanced attraction and following activation of effector cells from both the innate and the adaptive immune response. Monocytes were also highly attracted by the alternative DC and the high levels of HLA-DR expression in monocytes attracted within cancer patients suggest that they are not suppressive MDSC but could be functionally active antigen presenting cells able to further boost the effect of the vaccine DC.

## Abbreviations

alt-: alternative
DC: dendritic cells
GM-CSF: granulocyte macrophage colony-stimulating factor
IL: interleukin
IFN: interferon
mAb: monoclonal antibodies
MPLA: monophosphoryl lipid A
NK: natural killer
PBMC: peripheral blood mononuclear cells
PG: prostaglandin
poly IC: polyinosinic:polycytidylic acid
qPCR: quantitative real time PCR
RCC: renal cell carcinoma
SEB: staphylococcal enterotoxin B
TLR: toll like receptor
TNF: tumor necrosis factor
Treg: regulatory T cells
